# Ectopic Pregnancy
*A Paper read at a Meeting of the Bristol Medico-Chirurgical Society held in the University of Bristol on 12th November, 1930.


**Published:** 1931

**Authors:** R. S. Statham, H. L. Shepherd

**Affiliations:** Honorary Gynœcologist, Bristol Royal Infirmary; Lecturer in Obstetrics, University of Bristol; Obstetric Registrar, Bristol Royal Infirmary; Obstetric Tutor, University of Bristol


### ECTOPIC PREGNANCY.*

BY

R. S. Statham, M.D., Ch.M., F.C.O.G.,

*Honorary Gynæcologist, Bristol Royal Infirmary ;*
*Lecturer in Obstetrics, University of Bristol ;*

AND

H. L. Shepherd, M.B., Ch.M., M.C.O.G.,

*Obstetric Registrar, Bristol Royal Infirmary ;*
*Obstetric Tutor, University of Bristol.*

---

Ectopic pregnancy is of interest to most members of our profession. Its incidence is by no means rare, and the results depend so very largely for their success upon an early diagnosis that no apologies seem necessary for reporting this series of cases. The clinical material upon which this paper is based consists of 85 consecutive cases at the Bristol Royal Infirmary and 15 private cases. The latter were added as they included three of the most interesting cases in our series.

The frequency of occurrence of ectopic pregnancy has been assessed variously. Wynne[14] found it in 1·3 per cent. of 22,500 gynæcological patients admitted to The Johns Hopkins Hospital. Schumann[9] made an analysis of all the pregnancies reported in Philadelphia in one year, and then obtained figures from all the operators in the area, thus arriving at an estimated incidence of one ectopic in 300 pregnancies.

* A Paper read at a Meeting of the Bristol Medico-Chirurgical Society held in the University of Bristol on 12th November, 1930.

It is of some interest to note that up to 1876 this condition was considered a pathological curiosity, which had never been noted at many leading hospitals. But in 1883 Lawson Tait diagnosed and operated upon a case of tubal pregnancy and reported his result in a monograph,[11] which started a long series of papers upon this most interesting condition.

Ectopic pregnancy results from some factor causing delay, or arrest, in the passage of the ovum from the Graafian follicle to the uterine decidua. The following varieties can therefore occur, in theory at any rate: (1) Primary ovarian pregnancy, (2) primary abdominal implantation, (3) tubal implantation, (4) secondary abdominal or tubo-abdominal implantations, (5) broad ligament pregnancy, (6) pregnancy in an undeveloped horn of the uterus.

Of these primary ovarian pregnancy is very rare, and the very existence of primary abdominal pregnancy is still denied by most authorities, although a very convincing case has been recently reported, and Professor Rayner has kindly given me details of a case that he is about to publish, which seems quite beyond dispute.

### Causation.

Any factors which can cause delay in the passage of the fertilized ovum in its journey to the uterus may originate an ectopic pregnancy. These may be conveniently grouped as follows :—

A. *Congenital.*—(*a*) Persistent fœtal convolutions of the tubes. (*b*) Diverticula leading out of the tubal lumen.[3] (*c*) Accessory ostia, which do not communicate with the tube. These are said to be fairly common, but have very seldom been seen by us.

B. *Conditions resulting from previous attacks of salpingitis.*—This theory was based upon the undoubted fact that very many tubal pregnancies follow upon an attack of pelvic peritonitis, especially if it be gonococcal in origin. It was believed that the ciliary action of the tubal mucosa was destroyed and that the lumen became locally constricted, so that a spermatozoon could pass up but a developing ovum would be arrested on its way down. These ideas were shown to be erroneous, and Opitz[7] demonstrated the most likely causation. He showed that, as a result of previous salpingitis, the long tubal plicæ tend to become adherent in places and so form conical pockets with blind ends, into which the ovum may pass and become arrested.

C. Webster[12] advanced a theory that tubal pregnancy might be regarded as *an atavistic phenomenon,* basing it upon the decidual reaction in the tube, which he considered a return to the primitive type of uterus. But Bland Sutton states that ectopic pregnancy does not occur in the lower animals—though Blair Bell has described it in rats—and there is practically no material proof of this theory.

On the whole, the commonest cause of tubal pregnancy is probably the formation of tubal pockets following on salpingitis, and the presence of accessory ostia and congenital diverticula will account for a fair proportion of the remaining cases.

### Varieties.

The varieties of implantation in this series of 100 cases were as follows :—

| | |
|---|---|
| Primary ovarian pregnancy .. .. | 1 |
| Secondary abdominal pregnancy .. | 3 |
| Tubal implantation .. .. .. | 95 |
| Pregnancy in an undeveloped horn .. | 1 |

The specimen of *primary ovarian pregnancy* (No. 1) was a particularly fine one, in that the sac and embryo were quite intact. It has been reported in detail elsewhere. [2]

The patient had been treated for sterility of four years' duration by $CO_2$ insufflation, and became pregnant two months later. At 2½ months she developed typical symptoms of tubal pregnancy, and an immediate operation was performed, the ovary with the ectopic pregnancy being removed. She has since been delivered of a normal child.

The existence of primary ovarian pregnancy was strongly denied for many years by such eminent authorities as Tait and Bland Sutton, but in 1878 Spiegelberg laid down his four "rules" for the recognition of this condition, viz. (1) the tube on the affected side must be intact, (2) the fœtal sac must be in the position of the ovary, (3) the pregnancy must be connected to the uterus by the ovarian ligament, (4) true ovarian tissue must form its wall.

Since that date numerous cases have been recorded, and these include at least two in which pregnancy has terminated in the formation of lithopædia (Santti).

The three cases of *secondary abdominal pregnancy* were very interesting clinically.

The first patient (No. 2) was between five and six months pregnant, and showed all the symptoms of acute appendicitis. The temperature was 102° and the pulse-rate 112, while there was a definite mass like a hen's egg in the right iliac fossa. The fact that the pregnancy was very thin-walled was not noticed, and the case was diagnosed as pregnancy complicated by an appendix abscess or pyosalpinx. At operation the "mass" proved to be the fundus of the uterus pushed well back over to the right side by a thin-walled sac containing a fœtus. The operation was rendered very difficult by dense adhesions, but the placenta was luckily situated in the pouch of Douglas and was removed intact. The patient made an uneventful recovery, but the child survived only twenty minutes.

The second patient (No. 3) was a primigravida who had missed three periods, and was taken with a sudden attack of pain and vomiting. She came into Bristol, seven miles, in a side-car. She was found to have a biggish uterus with a mass the size of a grape-fruit in the pouch of Douglas, which was diagnosed as a pelvic hæmatocele. She refused operation as she had to go on haymaking, and this she did in spite of violent expostulations! Twelve days later she returned, and at the operation twin fœtuses were removed from the pouch of Douglas. The sac wall included the right tube and ovary; the blood-clot round the sac was already infected, but she made a perfectly normal recovery.

The third patient (No. 4) was admitted collapsed, and obviously had had an intraperitoneal hæmorrhage. There was a soft mass in the pouch of Douglas. When the abdomen was opened a fœtus and placenta were found lying on the back of the right broad ligament, and the placenta was firmly implanted into the back of the uterus and pouch of Douglas. The broad ligament contained old clot, and the case seems to have been firstly a tubal gestation, then a broad ligament pregnancy which re-escaped into the peritoneum, and gained a new implantation for the second time.

*True tubal pregnancy* is much the commonest type of ectopic gestation. The implantation can be interstitial, isthmic or ampullary. The average reported frequency is roughly interstitial 3 per cent., isthmic 20 per cent. and ampullary 77 per cent., and this series of cases approximates closely to these figures.

In any variety the fertilized ovum enters the tube, and is arrested during its passage to the uterus by one of the causes already discussed. The trophoblast then settles down, either on a plica, or on the actual wall of the tube between two plicæ, and then embeds itself by virtue of its penetrative power. The common site is "intercolumnar," or between two of the plicæ, so the ovum is very soon found lying in the muscularis of the tube, and surrounded by a capsule of fibrin from blood-clot and degenerated muscle tissue

(corresponding to Nitabuch's layer in a normal pregnancy), which separates it from the tubal lumen. In the rare cases of implantation on a plica the ovum will, of course, be entirely surrounded by the epithelial lining of the tube, and its area of expansion is then strictly limited. If the implantation takes place in the narrow isthmic portion of the tube early "rupture" will occur, but an ectopic pregnancy situated in the ampullary end may expand its capsule into the tube to a much greater extent before any severe symptoms supervene. A slight but definite decidual reaction occurs in the tube, and decidual cells have been found scattered over the capsule of the ectopic pregnancy in many cases, [13, 15] but no true decidua really exists as a definite structure.

The chorionic tissue and placental formation in the tube do not differ from those in intra-uterine gestation, with the exception of the fact that chorionic degeneration is common.[5] This was well seen in several of our cases, and the changes in the case of ovarian pregnancy (No. 1.) were fully studied and illustrated by Fraser in the report upon it.[2]

While these changes are taking place in the tube the uterus is also undergoing a characteristic reaction. Definite decidual formation occurs and has been very fully studied.[8] When fœtal death supervenes the decidua is expelled in small scraps accompanied by bleeding, but it may occasionally be passed in a complete uterine cast (Case No. 59).

### Termination.

A tubal pregnancy may terminate in a variety of ways: (*a*) formation of a tubal mole, (*b*) tubal abortion, (*c*) intraperitoneal rupture, (*d*) intraligamentary rupture.

The first two varieties (*a* and *b*) can be grouped together as "internal rupture of the capsule," while the second pair (*c* and *d*) represent "external rupture of the capsule."

(*a*) *Tubal mole.*—In this condition the blood from the maternal vessels—which have been eroded by the trophoblast—enters the lumen of the tube, while the ovum itself is not completely detached. Clotting then occurs round the ovum with resulting fibrosis, and this process being repeated at short intervals results in the formation of a mole. The mole is usually expelled into the peritoneal cavity at a later date, though occasionally fibroid remnants of a mole are found at operation which must have been in the tube for years.

(*b*) *Tubal abortion* is naturally most common in ampullary implantation, and consists of the expulsion of the ovum which has been *completely* detached by the blood. The ovum and blood are forced into the peritoneal cavity by the contractions of the intact portion of the tube. The colic and accompanying bleeding render it a matter of great difficulty to distinguish between tubal abortion and tubal rupture.

In some cases the blood will clot firmly round the fimbriæ of the tube and form a "peritubal hæmatoma."

(*c*) *Intraperitoneal rupture.*—In this condition the capsule of stretched tube, which encloses the ovum, is so eroded and thinned by the trophoblast that it gives way, and very severe bleeding may take place into the peritoneal cavity, which will produce all the classic symptoms of an abdominal catastrophe. In many cases the bleeding is not so severe, and the blood clots about the affected portion of the tube and produces the paratubal hæmatoma. In other cases clotting will take place in the pouch of Douglas, giving rise to a "pelvic hæmatocele." It must be noticed that the real cause of the bleeding is erosion, and not distension of the tube wall, and so the term "rupture"—long established in our nomenclature—is really a misnomer. As about four-fifths of the tubal surface is covered with peritoneum, it is natural to find this variety much commoner than the next, viz.:—

(*d*) *Intraligamentary rupture.*—Here the tube gives way at a point on its wall which is lying over the tissues of the broad ligament. The blood passes into the broad ligament and distends it, forming a "pelvic hæmatoma."

In addition to the above varieties there are three very rare terminations which are occasionally seen. In a few cases of intraperitoneal rupture the ovum may be reimplanted in the pelvis and continue to grow, and so produce a "secondary abdominal pregnancy," including the so-called "tubo-abdominal pregnancy." Three cases are here recorded (Nos. 2, 3 and 4).

The ovum in the case of intraligamentary rupture may reimplant itself within the layers of the broad ligament and develop in that situation. Both these varieties may go to full time; and viable, but usually deformed infants, have been removed at term. We have had no case of this at the Royal Infirmary.

Finally, a "lithopædion" may be formed, and be discovered years later. This condition seems to be much rarer than it used to be, probably owing to the great improvement in early diagnosis which has taken place.

In one of our cases (No. 100) the pregnancy occurred in a rudimentary horn, and gave rise to violent bleeding at the fourth month, although no symptoms at all had occurred till that time.

### Diagnosis.

There are obviously two very different conditions to be diagnosed as ectopic pregnancy, viz. (1) cases of severe intraperitoneal bleeding, and (2) those in which the loss of blood is slight, or has taken place into the tube or broad ligament only.

In the first class there is no difficulty in deciding that a very acute abdominal catastrophe has occurred. The patient is blanched, sweating, and cold. The pulse is thin and rapid. Free fluid can be demonstrated in the flanks, and usually there is well-marked air hunger. In these cases the diagnosis is fairly obvious, while there is never any question as to the urgent necessity of operative interference.

It is in the second group of cases that the difficulty of diagnosis arises. Wynne[14] states that at Johns Hopkins only 46 per cent. of cases were correctly diagnosed in 1919, while Brady[1] in 1923 found it had improved to over 70 per cent. in the same clinic. The correct diagnosis of an ectopic pregnancy is obviously of the greatest importance, and every case operated upon early can be regarded as having been saved from a grave disaster; for it is quite impossible to foresee the occurrence of a tubal mole or abortion in place of a tubal rupture.

In the first place, we regard the history of a missed period as of little importance. Brady[1] found it present in only 50 per cent. of his cases, and in this series one in six had not definitely missed any period at all, while in many more this history was doubtful. The complaint of irregular attacks of sharp pain of a *colic*

type is a much more important symptom, and when this is accompanied by irregular uterine bleedings—usually small in quantity—the diagnosis may be considered to be practically established on the history alone.

On pelvic examination it is usually possible to distinguish a mass which is very tender, to one side of the uterus, or in the pouch of Douglas, often associated with enlargement of the uterine artery of that side which can easily be felt pulsating in the fornix affected. The uterus itself will be enlarged and softened and suggests an early pregnancy. In some cases small clots of blood may be felt in the pouch of Douglas. The attacks of colic are due, of course, to the bleeding into the tube and small intraperitoneal hæmorrhages, while the vaginal bleeding is the result of the attempts at getting rid of the degenerating decidua, and occurs coincidently with the colic, due to the tubal bleeding which has killed the embryo. A sign which is sometimes of great value, and much vaunted by French gynæcologists, is the "rising pain," present in about 45 per cent. of this series. It consists of a dull ache passing up from the pelvis and running to the right shoulder, where it persists as a steady ache.

In our opinion any woman who has suffered with attacks of colic and irregular uterine hæmorrhage should be at once diagnosed as a suspect ectopic pregnancy, and be placed under close supervision. If no alternative and certain diagnosis be found, she should be treated by abdominal section without undue delay.

In a series of 29 cases at the Royal Infirmary, operated upon on the evidence of history only, we found 26 ectopic pregnancies, 1 pyosalpinx, 1 twisted ovarian cyst, and 1 normal pregnancy; an error rate in diagnosis of 10 per cent., but only 3·5 per cent. when the question of the need of an operation is considered, and this fully justifies the unnecessary operations.

There is one point which cannot be too heavily insisted upon, and that is the very real danger of vaginal examination in these cases, unless it is possible to open the abdomen at once.

One of the patients in this series (No. 30) was examined in the ward with great care, and almost at once complained of a severe pain. Within three minutes the pulse ran up to 120 and she became pale and collapsed. She was rushed into the theatre, and the abdomen was opened and the bleeding dealt with; but she lost quite 1½ pints of blood during the few minutes involved in getting her on to the table and under the anæsthetic. Also, a number of years ago, a case was admitted to a surgical ward at the Royal Infirmary and examined there in bed. The same events occurred, and the patient died before the abdomen could be opened, although there was only a few minutes' delay.

It seems desirable to make the tentative diagnosis of an ectopic pregnancy on the history, and to delay confirmation by vaginal examination till the patient is in hospital, or a nursing home, where she can be operated upon immediately, if necessary.

The chief *differential diagnosis* is from incomplete abortion. In this series of cases 56 were not obviously "immediates," and of these ten were actually diagnosed as "retained products" and curetted, *i.e.* an error of 18 per cent. The true diagnosis was made in eight of these cases while the patient was on the table, and the abdomen was immediately opened. In the two remaining cases (Nos. 63 and 97) the condition was not diagnosed and both patients died, being the only deaths in the series. It is of interest to note that one of these cases (No. 63) was both an ectopic and an intra-uterine pregnancy. We have found the most helpful differential points to be the fact that colic-like pain is very unusual in incomplete abortion, unless the uterus is actually in the process of expelling its contents, while the bleeding is very much more profuse than is usual in a tubal pregnancy. Also no swelling can be felt in the broad ligament or in the pouch of Douglas in the case of abortion. In cases where there is a large pelvic hæmatocele it is possible to mistake the swelling for a retroverted gravid uterus, and attempts to reduce it may have diasatrous results (No. 26). The hæmatocele may also be mistaken for a twisted ovarian cyst.

A case of abortion with lead colic was saved from operation as for ectopic gestation by the chance observation of a " blue line."

As an aid to diagnosis in very doubtful cases the introduction of an exploring needle into the pouch of Douglas may give valuable information.

### Treatment.

Operation is the only possible form of treatment, once the diagnosis has been made. Even if the symptoms point to a tubal abortion, it is much safer to open the abdomen immediately. Two of these were diagnosed as cases of tubal abortion by competent observers, and yet were found to be early tubal ruptures at operation, and might have bled violently at any minute.

In cases with slight bleeding it is our custom to remove the affected tube and retain the ovary when possible, and it is most important to inspect the other tube. Twin tubal pregnancy is not unduly rare, and three cases of triplets have been recorded. Also, the other tube may be so diseased as to require removal. The occurrence of an intra-uterine and tubal pregnancy at the same time is comparatively frequent. Novak[6] records 276 cases, and it occurs twice in our series (No. 50 and 63).

In one case in this series a second tubal pregnancy occurred in the stump of the tube removed at a previous operation for ectopic pregnancy (No. 34), a condition of which Hasselblatte[3] reports 21 cases. Four of this series had had previous tubal pregnancies on the other side (Nos. 7, 66, 68, 85).* This is quite a usual percentage. Smith[10] analysed 144 cases, and found that 80 (56 per cent.) became pregnant again. Of these 23 were ectopic (28·6 per cent), or 16 per cent. of the whole 144 cases. Other authorities give similar figures, so that Sampson and Smith consider it quite justifiable to remove both tubes at the first operation. This is not our custom.

In cases of ruptured tubal pregnancy with severe bleeding immediate operation is, of course, indicated, and whenever possible a blood transfusion should be given during the operation. This has a most dramatic effect, and undoubtedly saved the lives of three of our cases. Before the abdomen is opened two Carwardine intestinal clamps are placed ready. The abdomen is opened and the hand is at once plunged down into the pelvis, and the mass of clot and tube grasped and clamped on both sides, so as to stop the bleeding. This should *always* be done, as furious bleeding often begins as soon as the abdomen is opened, and it is useless to try and mop it away

* Since this paper was read on the 12th November another case of repeated tubal pregnancy has been operated upon at the Bristol Royal Infirmary, which is included in the list of cases as No. 13 on the first occasion.

before the clamps are applied. As it is not possible to see if the bowel is adherent to the sac, we use these light bowel clamps, which do no injury to any adherent gut which may be included in their grasp. The blood and clot are rapidly removed, for which we prefer a suction apparatus, and then the mass between the clamps is inspected and the tube and pregnancy removed. It is our habit to remove the clot and blood, for although it has been stated that the blood will be reabsorbed with benefit to the patient, we cannot but think it is a peritoneal irritant and forms an excellent nidus for sepsis. We have no experience of using the blood baled from the peritoneum for transfusion into the patient's vein, which is highly spoken of by many German authors. Rapidity of operation is essential, and immediate steps must be taken to combat shock. We find an electrically-heated blanket invaluable. A saline infusion is given as required.

We have had no experience of operating upon viable broad ligament pregnancies. Finally, a smear should be examined for gonorrhœa and a Wassermann test done during convalescence.

### Results.

Out of this series of 100 cases two deaths occurred, being 2 per cent. of the total. The remainder made a good recovery, and this is comparable with the collected figures of Schauta, which showed a death-rate of 5·7 per cent. in operated cases as against 87 per cent. in cases treated by expectant methods.

The deaths were as follows. Case No. 63 had an intra-uterine abortion, badly infected, as well as a tubal pregnancy.

Peritonitis followed the curetting. Laparotomy three days later was followed next day by death from general peritonitis. Case No. 97 was diagnosed as "retained products." The temperature was 101° on admission, curetting was followed by acute peritonitis, and on laparotomy twenty-four hours later a necrotic broad ligament pregnancy was found. Death followed from general peritonitis.

On the *diagnosed* cases, therefore, the mortality was nil.

### SUMMARY.

In summarizing our series of 100 cases we have been faced with one great difficulty, viz. the classification of those cases which do not show a definite tubal rupture. These cases rank high in our series owing to early diagnosis. The condition found showed a thin tube distended with a blood-clot, but no definite gap in the tube wall. Although many of these would have eroded through in a short time, we have thought it advisable to classify them as "tubal abortion," as the far preferable term "intratubal rupture," advocated by Berkeley and Bonney, is not in general use.

*Total cases*, 100.—Died, 2 = 2 per cent; recovered, 98 = 98 per cent.

*Site of Ectopic Pregnancy.*—Ovarian, 1; abdominal, 3; tubal, 88 (intestinal, 4; isthmic, 3; ampullary, 80; stump, 1); broad ligament, 7; rudimentary horn, 1.

*Termination.*—Abortion, 45; mole, 5; rupture, 45 (intraperitoneal, 38; broad ligament, 7).

*Clinical aspects on admission.*—Collapsed, 32; not collapsed, 63.

Chief symptoms in not collapsed cases: pain, 55; hæmorrhage, 43; tumour, 26.

*Previous ectopic pregnancies.*—4 cases.

| *Age.* | | | *Parity.* | |
|---|---|---|---|---|
| 20 years .. | 1 case | | 0 .. | 25 cases. |
| 21–30 ,, .. | 41 cases | | 1 .. | 32 ,, |
| 31–40 ,, .. | 34 ,, | | 2 .. | 13 ,, |
| 41 ,, or over | 4 ,, | | 3 .. | 13 ,, |
| Not stated .. | 20 ,, | | 4 .. | 7 ,, |
| | | | 5 and over | 8 ,, |
| | | | Not stated | 2 ,, |

| *Years since last delivery.* | | | *Weeks since last regular period.* | |
|---|---|---|---|---|
| 1 year .. | 10 cases. | | 1–2 .. | 4 cases. |
| 2 years .. | 10 ,, | | 3–4 .. | 18 ,, |
| 3 ,, .. | 11 ,, | | 5–6 .. | 20 ,, |
| 4 ,, .. | 5 ,, | | 7–8 .. | 25 ,, |
| 5 ,, .. | 8 ,, | | 9–10 .. | 5 ,, |
| 6 ,, .. | 3 ,, | | 11–12 .. | 12 ,, |
| 7 ,, or more | 15 ,, | | 13–16 .. | 5 ,, |
| Not stated .. | 13 ,, | | 17–20 .. | 1 case. |
| | | | 20 and over | 3 cases. |
| | | | Lactating | 2 ,, |
| | | | Not stated | 5 ,, |

| Number. | Age. | Parity. | Years since last delivery. | Weeks since last regular period. | Symptoms. Pain. | Hæmorrhage. | Tumour. | Collapse. | Side. | Type. | | Days in Hospital. | Result. |
|---|---|---|---|---|---|---|---|---|---|---|---|---|---|
| PRIMARY OVARIAN. | | | | | | | | | | | | | |
| 1 | 23 | 0 | — | 10 | ++ | 0 | 0 | 0 | R. | — | Tubes insufflated four months previously. | 20 | A. |
| SECONDARY ABDOMINAL. | | | | | | | | | | | | | |
| 2 | 27 | 0 | — | 22 | + | 0 | + | 0 | — | — | Child lived twenty minutes. | 21 | A. |
| 3 | 37 | 0 | — | 12 | + | 0 | + | 0 | R. | — | Placenta on right broad ligament. | 50 | A. |
| 4 | 38 | 3 | 5 | 9 | 0 | 0 | + | + | R. | — | Broad ligament pregnancy ruptured into peritoneal cavity and placental tissue and living fœtus in pouch of Douglas. | 19 | A. |
| TUBAL PREGNANCY.—IMMEDIATE OPERATIONS. | | | | | | | | | | | | | |
| 5 | ? | 3 | 9 | 5 | + | + | + | ++ | R. | Rupture | Intravenous saline .. | 30 | A. |
| 6 | 28 | 0 | — | 7 | + | + | + | + | L. | Rupture | — | 16 | A. |
| 7 | 30 | 0 | — | 12 | + | 0 | 0 | + | L. | Rupture | Previous ectopic two years ago at Hull. Interstitial also interuterine pregnancy. | 21 | A. |
| 8 | 26 | 1 | $1\frac{1}{2}$ | 8 | ++ | + | + | ++ | R. | Rupture | Isthmic .. .. .. .. | 29 | A. |
| 9 | 26 | 1 | 8 | 8 | + | + | + | 0 | L. | Rupture | — | 20 | A. |
| 10 | 33 | 1 | 7 | 24? | + | + | 0 | 0 | L. | Rupture | — | 26 | A. |
| 11 | 36 | 8 | $\frac{5}{12}$ | ? | + | + | 0 | 0 | L. | Rupture | Right tube ligated also | 18 | A. |
| 12 | 32 | 0 | — | 7 | + | 0 | 0 | + | R. | Rupture | — | 17 | A. |
| 13 | ? | 0 | — | ? | + | + | 0 | ++ | R. | Rupture | Blood transfusion .. | 29 | A. |
| 14 | 27 | 2 | ? | ? | 0 | 0 | 0 | ++ | L. | Rupture | Intravenous saline .. | 14 | A. |
| 15 | 36 | 3 | ? | 12 | + | 0 | + | + | R. | Rupture | — | 24 | A. |
| 16 | 36 | 3 | ? | 5 | + | + | 0 | 0 | R. | Rupture | — | 17 | A. |
| 17 | 33 | 1 | ? | 9 | + | 0 | 0 | ++ | ? | Rupture | Intravenous saline, *vide* No. 68. | 29 | A. |
| 18 | 25 | 1 | 8 | 10 | + | + | 0 | + | R. | Rupture | — | 21 | A. |
| 19 | 30 | 2 | $\frac{5}{12}$ | * | + | 0 | 0 | + | R. | Rupture | *Lactating .. .. .. | 13 | A. |
| 20 | 29 | 2 | 5 | 3 | + | 0 | 0 | 0 | R. | Rupture | Tubo-ovarian pregnancy | 15 | A. |
| 21 | 28 | 3 | 2 | 8 | + | + | 0 | ++ | ? | Rupture | — | 23 | A. |
| 22 | 29 | 0 | — | 12 | + | 0 | 0 | + | R. | Rupture | — | 14 | A. |
| 23 | 38 | 2 | ? | 5 | + | + | 0 | + | L. | Rupture | — | 21 | A. |
| 24 | ? | 1 | 9 | 7 | ++ | 0 | 0 | + | L. | Rupture | Intravenous and saline | 18 | A. |
| 25 | 25 | 1 | 4 | 2 | + | 0 | 0 | + | R. | Rupture | — | 28 | A. |
| 26 | 39 | 3 | 7 | 6 | + | + | 0 | + | R. | Rupture | Ruptured when retroversion was reduced. | 14 | A. |

| Number. | Age. | Parity. | Years since last delivery. | Weeks since last regular period. | Symptoms. Pain. | Symptoms. Hæmorrhage. | Symptoms. Tumour. | Symptoms. Collapse. | Side. | Type. | | Days in Hospital. | Result. |
|---|---|---|---|---|---|---|---|---|---|---|---|---|---|
| 27 | ? | 2 | 2½ | 6 | + | 0 | 0 | ++ | ? | Rupture | Puerperal sepsis at last confinement. | 21 | A. |
| 28 | 23 | 0 | — | 4 | + | + | 0 | 0 | L. | Rupture | Gonorrhœa .. .. .. | 21 | A. |
| 29 | ? | 0 | — | 14 | + | 0 | 0 | ++ | ? | Rupture | — | 20 | A. |
| 30 | 27 | 1 | 1½ | 5 | + | + | + | + | R. | Rupture | Ruptured during vaginal examination. | 21 | A. |
| 31 | 32 | 3 | 1½ | 8 | + | 0 | + | 0 | R. | Rupture | Interstitial .. .. .. | 18 | A. |
| 32 | 22 | 0 | — | 8 | + | 0 | 0 | + | R. | Rupture | Interstitial .. .. .. | 28 | A. |
| 33 | 37 | 3 | 10 | 8 | + | ++ | 0 | ++ | R. | Rupture | Isthmic .. .. .. .. | 19 | A. |
| 34 | 22 | 0 | — | 4 | + | 0 | 0 | ++ | R. | Rupture | In stump of tube removed at previous operation 6 months before. | 28 | A. |
| 35 | 26 | 1 | 4 | 3 | + | + | 0 | + | L. | Rupture | — | 12 | A. |
| 36 | 31 | 4 | 3 | 13 | + | + | 0 | ++ | R. | Mole | — | 19 | A. |
| 37 | 29 | 3 | 7 | 2 | + | ++ | 0 | + | R. | Abortion | — | 29 | A. |
| 38 | 31 | 0 | — | 6 | + | 0 | 0 | + | R. | Abortion | — | 44 | A. |
| 39 | 39 | 0 | — | 7 | ++ | 0 | 0 | + | R. | Abortion | — | 16 | A. |
| 40 | 33 | 6 | 5 | 8 | + | + | 0 | 0 | L. | Abortion | *Vide* No. 66 .. .. | 17 | A. |
| 41 | ? | 0 | — | 3 | + | + | 0 | ++ | L. | Abortion | — | 16 | A. |
| 42 | 31 | 1 | 5 | 3 | + | 0 | 0 | + | L. | Abortion | — | 18 | A. |
| 43 | 26 | 1 | 4 | 4 | + | ++ | 0 | + | L. | Abortion | — | 11 | A. |
| 44 | ? | 2 | ? | 5 | + | + | 0 | 0 | ? | Abortion | — | 21 | A. |

TUBAL PREGNANCY.—OPERATION OF ELECTION.

| Number. | Age. | Parity. | Years since last delivery. | Weeks since last regular period. | Pain. | Hæmorrhage. | Tumour. | Collapse. | Side. | Type. | | Days in Hospital. | Result. |
|---|---|---|---|---|---|---|---|---|---|---|---|---|---|
| 45 | ? | 4 | 10 | 8 | + | + | + | 0 | R. | Abortion | — | 20 | A. |
| 46 | ? | 5 | 1 | * | 0 | + | + | 0 | R. | Abortion | * Lactating. Broncho-pneumonia. | 62 | A. |
| 47 | 22 | 0 | — | 2 | + | + | 0 | 0 | R. | Abortion | Septic hæmatocele .. | 56 | A. |
| 48 | 29 | 1 | $\frac{5}{12}$ | 7 | 0 | ++ | + | 0 | L. | Abortion | Right tube ligated also | 25 | A. |
| 49 | 29 | 2 | 6 | 8 | + | + | + | 0 | L. | Abortion | Right hydro-salpinx removed. | 20 | A. |
| 50 | ? | 4 | 7 | 8 | + | + | + | 0 | R. | Abortion | A $\frac{5}{12}$ pregnancy in uterus | 25 | A. |
| 51 | ? | 1 | 5 | ? | 0 | + | + | 0 | L. | Abortion | — | 18 | A. |
| 52 | 25 | 0 | — | 8 | + | + | + | 0 | L. | Abortion | — | 20 | A. |
| 53 | 38 | 4 | $\frac{9}{12}$ | ? | + | + | + | 0 | R. | Abortion | Was curetted first, but diagnosed on the table. | 32 | A. |
| 54 | ? | ? | | 14 | + | + | 0 | 0 | L. | Abortion | Was curetted first, laparotomy 5 days later. | 25 | A. |
| 55 | 23 | 1 | $\frac{4}{12}$ | 3 | + | + | + | 0 | L. | Abortion | — | 29 | A. |
| 56 | ? | 1 | $\frac{9}{12}$ | 10 | + | + | + | 0 | R. | Abortion | Curetted first, but diagnosed on table. | 21 | A. |
| 57 | 32 | 1 | 2 | 7 | + | 0 | + | 0 | R. | Abortion | — | 17 | A. |
| 58 | 27 | 0 | — | 24 | + | + | 0 | 0 | L. | Abortion | — | 24 | A. |
| 59 | ? | 1 | 3 | 12 | + | ++ | 0 | 0 | L. | Abortion | Right tube ligated also. Passed a uterine cast. | 15 | A. |
| 60 | 30 | 2 | 3 | 7 | + | + | + | 0 | R. | Abortion | Right parovarian cyst .. | 18 | A. |
| 61 | 32 | 1 | ? | 6 | + | + | 0 | 0 | R. | Abortion | Appendicectomy also .. | 19 | A. |
| 62 | 34 | 1 | 7 | 3 | + | + | 0 | 0 | R. | Abortion | *Vide* No. 85 .. .. .. | 14 | A. |

| *Number.* | Age. | Parity. | Years since last delivery. | Weeks since last regular period. | Symptoms. Pain. | Hæmorrhage. | Tumour. | Collapse. | Side. | Type. | | Days in Hospital. | Result. |
|---|---|---|---|---|---|---|---|---|---|---|---|---|---|
| 63 | 33 | 1 | ? | 6 | + | + | 0 | 0 | L. | Abortion | (1) Curetted and removal of septic intra-uterine abortion. (2) Peritonitis 3 days later—laparotomy and removal of septic tubal abortion. Drainage. | 4 | D. |
| 64 | 29 | 1 | ? | 12 | 0 | + | + | 0 | R. | Abortion | Curetted, but diagnosed on table. Left hæmatosalpinx also removed. | 18 | A. |
| 65 | 34 | 1 | 4 | 6 | + | + | + | 0 | R. | Abortion | — | 17 | A. |
| 66 | 36 | 6 | 3 | 9 | + | + | 0 | 0 | R. | Abortion | *Vide* No. 40. Previous ectopic. | 14 | A. |
| 67 | 34 | 2 | 9 | 5 | 0 | + | 0 | 0 | L. | Abortion | Curetted 3 weeks previously for chronic uterine sepsis. Subtotal hysterectomy. | 23 | A. |
| 68 | 35 | 1 | 3 | 6 | + | 0 | 0 | 0 | L. | Abortion | *Vide* No. 17. Previous ectopic. | 24 | A. |
| 69 | 33 | 0 | — | 4 | + | 0 | 0 | 0 | R. | Abortion | Left ovarian. Double salpingectomy. | 14 | A. |
| 70 | 24 | 1 | 1½ | 3 | + | + | 0 | 0 | R. | Abortion | — | 11 | A. |
| 71 | 34 | 1 | 10 | 16 | + | 0 | 0 | 0 | L. | Abortion | — | 18 | A. |
| 72 | 29 | 2 | 1¾ | 4 | + | + | 0 | 0 | L. | Abortion | Curetted, diagnosed on table. | 35 | A. |
| 73 | 42 | 7 | 3 | 5 | + | + | 0 | 0 | R. | Abortion | Left ovarian dermoid .. | 31 | A. |
| 74 | 39 | 4 | 5 | 12 | 0 | + | 0 | 0 | R. | Abortion | Curetted and diagnosed on table. | 30 | A. |
| 75 | 17 | 0 | — | 4 | + | 0 | 0 | 0 | L. | Abortion | — | 21 | A. |
| 76 | 32 | 4 | 2 | 12 | + | + | 0 | 0 | L. | Abortion | — | 17 | A. |
| 77 | ? | 3 | ? | 4 | + | 0 | 0 | 0 | R. | Abortion | — | 21 | A. |
| 78 | ? | 0 | — | 3 | + | + | 0 | 0 | R. | Abortion | — | 21 | A. |
| 79 | 43 | 6 | 7 | 6 | + | + | + | 0 | L. | Abortion | — | 16 | A. |
| 80 | 28 | 1 | 1 | 4 | + | 0 | 0 | 0 | R. | Abortion | — | 21 | A. |
| 81 | 44 | 0 | — | 6 | + | + | 0 | 0 | L. | Abortion | Polypus extruding through cervix, hysterectomy. | 18 | A. |
| 82 | 24 | 1 | 2 | 5 | + | + | + | 0 | L. | Rupture | Small left ovarian .. | 22 | A. |
| 83 | 28 | 2 | 1 | 2 | + | + | ++ | 0 | R. | Rupture | — | 23 | A. |
| 84 | 28 | 1 | 4 | 7 | + | 0 | 0 | 0 | L. | Rupture | Appendicectomy .. .. | 17 | A. |
| 85 | 36 | 3 | 3 | 6 | + | 0 | 0 | 0 | L. | Rupture | Previous ectopic, *vide* No. 62. | 18 | A. |
| 86 | 25 | 4 | 7/12 | 6 | + | 0 | 0 | 0 | R. | Rupture | — | 21 | A. |
| 87 | ? | 3 | ? | ? | + | + | 0 | 0 | R. | Rupture | — | 18 | A. |
| 88 | 31 | 7 | 6 | 12 | + | 0 | + | 0 | R. | Rupture | — | 22 | A. |
| 89 | ? | 1 | 3 | ? | 0 | + | + | 0 | L. | Mole | — | 18 | A. |
| 90 | 30 | 1 | 8 | 3 | + | + | 0 | 0 | R. | Mole | Curetted and diagnosed on table. | 23 | A. |
| 91 | 26 | 0 | — | 6 | 0 | + | 0 | 0 | L. | Mole | Right tube ligated also | 20 | A. |
| 92 | 26 | 2 | 2 | 8 | + | + | + | 0 | L. | Mole | — | 17 | A. |

| Number. | Age. | Parity. | Years since last delivery. | Weeks since last regular period. | Symptoms. Pain. | Hæmorrhage. | Tumour. | Collapse. | Side. | Type. | | Days in Hospital. | Result. |
|---|---|---|---|---|---|---|---|---|---|---|---|---|---|
| | | | | | | | | | | | BROAD LIGAMENT PREGNANCIES. | | |
| 93 | 35 | 8 | ¾ | 12 | + | + | + | ++ | R. | Broad Ligament | Secondary rupture into peritoneum. Intravenous saline. | 28 | A. |
| 94 | ? | 1 | ? | ? | + | 0 | + | 0 | R. | Broad Ligament | — | ? | A. |
| 95 | ? | 1 | 6 | 3 | + | + | + | 0 | L. | Broad Ligament | — | 21 | A. |
| 96 | 30 | 2 | 5 | ? | + | + | 0 | 0 | L. | Broad Ligament | — | 23 | A. |
| 97 | 43 | 3 | 2½ | 12 | + | + | + | 0 | L. | Broad Ligament | Curetted as intra-uterine abortion. 2 days later acute peritonitis. Gas, pus, and macerated fœtus in broad ligament. | 5 | D. |
| 98 | 36 | ? | ? | 8 | + | + | ++ | 0 | R. | Broad Ligament | Appendix removed .. | 24 | A. |
| 99 | 27 | 0 | — | 11 | + | + | 0 | 0 | R. | Broad Ligament | — | 16 | A. |
| | | | | | | | | | | | PREGNANCY IN RUDIMENTARY HORN. | | |
| 100 | 27 | 1 | 3 | 16 | + | 0 | 0 | ++ | L. | Rupture | Ruptured left rudimentary horn with abdomen full of blood. | 11 | A. |

### REFERENCES.

[1] Brady, *Johns Hopkins Hosp. Bull*, 1923, xxxiv. 152.
[2] Fraser & Statham, *Jour. Obst. and Gyn. Brit. Emp.*, 1927, xxxiv. 788.
[3] Hasselblatte, *Acta Obst. et Gyn. Scandin.*, 1927, vi. 211.
[4] McNally, *Amer. Jour. Obstet.*, 1926, xii. 303.
[5] Meyer, *Surg. Gyn. and Obstet.*, 1919, xxviii. 293.
[5] Meyer, Contribution to *Embryology*, 1920, ix. 327.
[6] Novak, *Surg. Gyn. and Obstet.*, 1926, xliii. 26.
[7] Opitz, *Ztschr. f. Geb. u Gyn. Stuttg.*, 1902-3, xlviii. 538.
[8] Sampson, *Trans. Amer. Gyn. Soc.*, 1913, xxxviii. 121.
[9] Schumann, *Extra-uterine Pregnancy*, Monograph, 1921.
[10] Smith, *Surg. Gyn. and Obstet.*, 1914, xviii. 684.
[11] Tait, Lecture on Ectopic Pregnancy, 1888.
[12] Webster, *Ectopic Pregnancy*, 1895.
[13] Williams, Whitridge, *Obstetrics*, 1930, p. 789.
[14] Wynne, *Johns Hopkins Hosp. Bull.*, 1919, xxx. p. 15.
[15] Young, *Edin. Med. Jour.*, 1909, iii. 118.

